# Digital zoom of the full-field digital mammogram versus magnification mammography: a systematic review

**DOI:** 10.1007/s00330-020-06798-6

**Published:** 2020-03-28

**Authors:** Mona Øynes, Bergliot Strøm, Bente Tveito, Bjørg Hafslund

**Affiliations:** 1 Department of Health and Functioning, Faculty of Health and Social Sciences, Western Norway University of Applied Sciences, Høgskulen på Vestlandet, Postbox 7030, 5020 Bergen, Norway; 2 Division of Research, Internationalisation and Innovation, Library, Western Norway University of Applied Sciences, Høgskulen på Vestlandet, Postbox 7030, 5020 Bergen, Norway

**Keywords:** Mammography, Diagnostic test, routine, Phantoms, imaging, Calcinosis

## Abstract

**Objectives:**

To summarise and compare the performance of magnification mammography and digital zoom utilising a full-field digital mammography (FFDM) system in the detection and diagnosis of microcalcifications.

**Methods:**

We ran an extended search in MEDLINE, EMBASE, CINAHL, Engineering Village and Web of Science. Diagnostic test studies, experimental breast phantom studies and a Monte Carlo phantom study were included. A narrative approach was selected to summarise and compare findings regarding the detection of microcalcifications, while a hierarchical model with bivariate analysis was used for the meta-analysis of sensitivity and specificity for diagnosing microcalcifications.

**Results:**

Nine studies were included. Phantom studies suggested that the size of microcalcifications, magnification or zoom factor, exposure factors and detector technology determine whether digital zoom is equivalent to magnification mammography in the detection of microcalcifications. Pooled sensitivity for magnification and zoom calculated from the diagnostic test studies was 0.93 (95% CI 0.84–0.97) and 0.85 (95% CI 0.70–0.94), respectively. Pooled specificity was 0.55 (95% CI 0.51–0.58) and 0.56 (95% CI 0.50–0.62), respectively. The differences between the sensitivities and specificities were not statistically significant.

**Conclusions:**

Digital zoom may be equivalent to magnification mammography. Diagnostic test studies and phantom studies using newer detector technology would contribute additional knowledge on this topic.

**Key Points:**

*• The performance of digital zoom is comparable to magnification for detecting microcalcifications when newer detector technology and optimised imaging procedures are utilised.*

*• The accuracy of digital zoom appears equivalent to geometric magnification in diagnosing microcalcifications.*

**Electronic supplementary material:**

The online version of this article (10.1007/s00330-020-06798-6) contains supplementary material, which is available to authorized users.

## Introduction

Microcalcifications in the breast can be challenging to diagnose [1, 2]. Until now magnification mammography has been required in diagnostic mammography units [3]. Nowadays, modern equipment with digital zooming is also used [4] in the diagnosing process. This paper seeks to elucidate whether choosing one technique over the other makes any differences when detecting and diagnosing microcalcifications.

Magnification mammography, hereafter referred to as ‘magnification’, is commonly used as complementary imaging on suspicion of microcalcifications. Complementary imaging decreases sensitivity and increases specificity [5] preventing unnecessary biopsies of benign lesions [5, 6].

Increased contrast-to-noise ratio (CNR) between microcalcifications and surrounding tissue, signal-to-noise ratio (SNR) and spatial resolution improves the visual conception of microcalcifications [7–12]. It is worth noting that the following factors can significantly affect the values of these quantities: absorption characteristics of the detector, detector pixel size and depth, focus size, monitor size, monitor pixel size and depth, properties of the X-ray spectrum, detector dose, properties of irradiated objects and removal of scattered radiation from the object. The use of post-processing algorithms also affects the image quality [13, 14].

The larger breast detector distance utilised in magnification leads to reduced effective pixel size, and when combined with smaller focus size, it yields better spatial resolution compared with conventional FFDM [15, 16]. However, studies show that average glandular dose (AGD) when using magnification is about twice that of breast imaging without magnification [17–19]. Digital zoom of conventional FFDM, hereinafter referred to as ‘zoom’, is a post-processing method that does not increase the AGD, nor does it improve spatial resolution [4].

When women are recalled due to suspicion of microcalcifications, the use of magnification leads to more image uptakes with potentially painful compression and longer examination time. This raises the question of whether zoom could replace magnification without leading to more undetected microcalcifications while reducing sensitivity and specificity. The added value would be fewer painful compressions and a reduction in AGD. It could also streamline workflow [20].

The aim of this study is to review the literature to:

- Summarise and compare the ability to detect microcalcifications utilising magnification and zoom.
- Summarise and compare the sensitivity and specificity of diagnosing microcalcifications utilising magnification and zoom in connection with recall due to suspicion of microcalcifications.

## Method

This study follows the guidelines of the Preferred Reporting Items for Systematic Reviews and Meta-Analyses (PRISMA) [21]. The protocol is registered in PROSPERO [22], registration number CRD42017057193.

### Literature search strategy

A computerised search was performed to identify original studies on detecting or diagnosing microcalcifications utilising magnification, zoom or both. The studies included were located by searching MEDLINE (Ovid), EMBASE (Ovid), CINAHL (EBSCO), Engineering Village: Compendex and Web of Science (last search date 10.09.2019). The literature search included controlled vocabulary terms and free-text terms in the following combination: (mammography OR microcalcification) AND (digital magnification OR geometric magnification).

There were no restrictions on language or publication dates. Reference lists of included articles were screened for additional references. Abstracts and posters from relevant conferences and grey literature databases were also screened. (The search strategy is described in detail in Electronic Supplementary Material.)

All references were exported to Endnote [23] for duplicate removal. Rayyan [24] was used for study selection.

### Study selection

Inclusion criteria: (1) Experimental studies with physical or Monte Carlo simulated phantoms of digital zoom or magnification mammograms or both focusing on microcalcifications. (2) Studies of mammograms from non-symptomatic women recalled after screening for diagnostic mammography where zoom, magnification or both were used or compared for diagnosis of microcalcifications. Exclusion criteria: (1) studies based on analogue film-screen, computed radiography (CR) mammograms, print-out/hard copies of digital mammograms, other modalities than FFDM and computer-aided detection/diagnostics; (2) studies based on imaging palpable tumours; (3) studies with patients with previous cancer disease or BRAC1/BRAC2; (4) studies with male patients, animals or specimens; (5) case reports, review articles, editorials, letters, consensus statements and studies focusing on cost.

Two reviewers (M.Ø. and B.S.) synchronised 20 randomly selected articles before they independently reviewed the titles and abstracts against the inclusion criteria and exclusion criteria. Any disagreement over the eligibility of particular studies was resolved by consensus. The full text of these studies was retrieved and independently assessed for eligibility. Any disagreement was resolved through discussion until the reviewers reached consensus.

### Data extraction and quality assessment

The two reviewers extracted relevant data from the studies included. Standardised data forms were used: (a) study characteristics: authors, year of publication, study period, affiliation and study design; (b) clinical characteristics: number of readers and their level of experience, diagnostics scale and threshold, pre-test probability, case characteristics and reference standard, number of cases, patient age and numbers of true and false positives and negatives; (c) technical characteristics: type of detector technology, pixel size and depth, exposure factors, magnification and zoom factors, focal spot size, monitor size and depth, characteristics of phantoms and outcome measures for phantom studies.

The two reviewers independently assessed the methodological quality of included studies. For diagnostic studies, QUADAS 2 [25] was used. For phantom experiment studies, risk of bias and applicability were assessed using an adapted version of QUADAS 2, where the ‘patient selection’ domain was replaced with questions about controlling confounding variables and the reference standard domain was omitted. Disagreements between the reviewers were resolved by discussion and consensus.

### Data synthesis and analysis

The outcomes of this systematic review were detection of microcalcifications, and sensitivity and specificity for diagnosing microcalcifications from images obtained with magnification techniques or using zoom. To assess the detection of microcalcifications, results from the phantom studies were used, while diagnostic performance was assessed from the results of the diagnostic test studies. Analysis of detection and diagnosis was performed separately.

Different measures of detectability were expected. The authors therefore decided to draw up a narrative explanation to summarise and compare findings regarding the detection of microcalcifications, rather than calculating pooled values.

To assess the sensitivity and specificity, the hierarchical model for meta-analysis of sensitivity and specificity with bivariate analysis was used [26–28]. Numbers of true positives, false negatives, false positives and true negatives from the diagnostic test studies were entered in the calculations. Sensitivities and specificities of the individual studies as well as the pooled sensitivity and specificity were calculated and presented in forest plots.

Heterogeneity among studies included in the meta-analysis was assessed using both the Cochrane *Q* test [29], where *p* < 0.05 indicates the presence of heterogeneity, and the inconsistency index (*I*^2^) [30]. *I*^2^ = 0–40% means heterogeneity might not be important; 30–60% moderate heterogeneity; 50–90% substantial heterogeneity and 75–100% considerable heterogeneity [31].

There were too few studies to perform a test of publication bias using a funnel plot [32].

The module ‘midas’ [33] and the built-in function ‘xtmelogit’ in Stata 15.1 [34] were used for the statistical analysis. The use of ‘xtmelogit’ is based entirely on the tutorial of Takwoingi [35]. A *p* value < 0.05 was considered statistically significant.

## Results

### Study selection

**Fig. 1:**
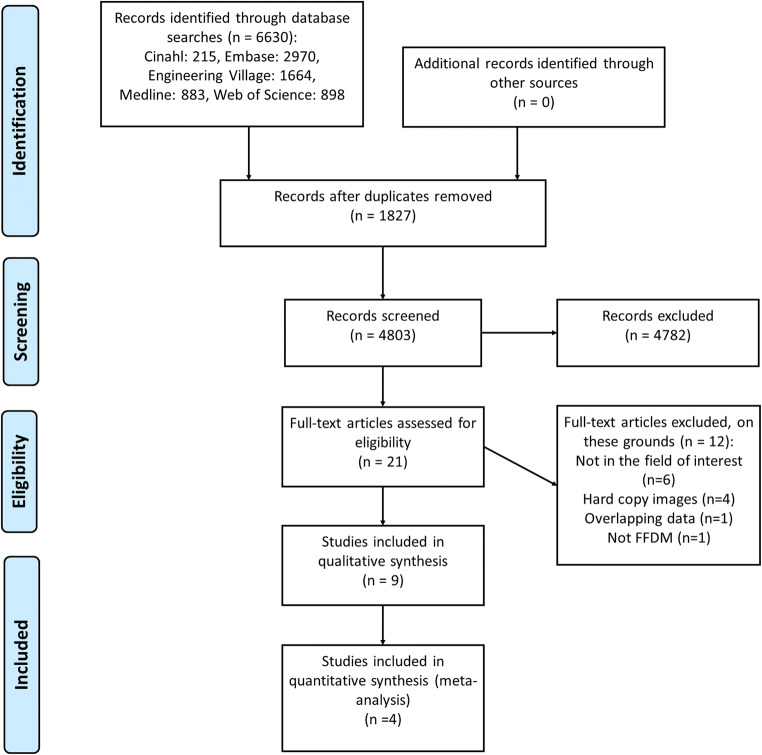
Flowchart (PRISMA diagram) of the study selection process

A flowchart of the study selection was generated as a PRISMA [21] diagram included here as Fig. 1. The initial search found 6630 articles. Search in grey literature yielded no additional articles. A total of 1827 articles were identified as duplicate, and the remaining title and abstracts were screened for inclusion and exclusion. This process whittled the total down to 21 articles to be read in full by two reviewers, and 4782 to be excluded.

Following the close reading, further 12 articles were excluded and 9 articles were finally selected for inclusion: five experimental phantom studies [36–40] and four retrospective diagnostic test studies [41–44].

### Characteristics of included studies

Study characteristics, number of readers, experience of readers and technical characteristics of experimental phantom studies are listed in Table 1. Monitor size was 5 megapixels, focus spot size for magnification 0.1 mm and zoom 0.3 mm for the included studies. Readers were allowed to adjust window width and window levels of the images in two studies [36, 38]. Otherwise, post-processing algorithms other than zoom were not mentioned in the studies.

**Table 1:** Experimental phantom studies: study characteristics, clinical characteristics and technical characteristics

Author	Year	Location	No. of readers	Reader experience, years/mean/SD/ range	Apparatus/detector technology	Pixel size/pixel depth	Effective pixel size magnification	Zoom factor	Magnification factor	Target/filter material	Current-time product (mAs)	Tube potential (kVp)	Phantom/simulated composition	Phantom thickness/simulated glandularity	Size of simulated microcalcifications/disks	Outcome measures
Alkhalifah et al [36]	2016	Kuwait	5	15/NS/NS	GE Senographe DS/indirect CsI scintillator detector	100 μm	63 μm	1.5	1.6	Mo/Mo Mo/Rh Rh/Rh	AEC	Range 26 kVp-32 kVp at intervals of 2 kVp	ACR [45]/ Al_2_O_3_ specs, diameter 0.16–0.54 mm	4.5 cm/ 50%	0.16–0.54 mm (diameter)	Rank sum scores and mean scores for visibility of microcalcifications
Egan et al [37]	2012	Ireland	NA*	NA*	Philips MDM/Photon counter Hololic Selenia/direct amorphous Selenium detector Ge Seno Essential/indirect CsI scintillator detector	50 μm 70 μm 100 μm	NA 39 μm 56 μm	A preset zoom NU NU	NA 1.8 1.8	W/Al W/Rh W/Ag Mo/Mo Mo/Rh Rh/Rh	AEC and manually chosen value to obtain a constant reference pixel value	Range 24 kVp-36 kVp at intervals of 2 kVp	Al square of 0.2-mm thickness embedded in PMMA	4, 5 and 6 cm/ NS	Al square of 0.2 mm thickness	Normalised PI PI = CNR^n^/AGD
Vaheyn et al [38]	2012	Australia	2	NS/NS/NS	GE Senographe DS/indirect CsI scintillator detector	100 μm	56 μm	1.8	1.8	Rh/Rh	AEC	Zoom: 29 kVp Mag.: 31 kVp	CDMAM [46]/ gold disks, diameters 0.13–2 mm	5 cm/NS	0.13–2.00 mm (diameter)	IQF and CDD
Koutalonis et al [39]	2010	UK	NA*	NA*	Monte Carlo model	50 μm	50 μm	Range 1.0–2.0, at intervals of 0.1	Range 1.0–2.0 at intervals of 0.1	Mo/Mo	AEC	28 kVp	Monte Carlo model [47]/ CO and HA radii 0.05–0.75 mm	4 cm / Range 10% -90% at intervals of 10%	0.05–0.75 mm (diameter)	CNR
Hermann et al [40]	2002	Germany	3	NS/NS/NS	GE Senographe DS/indirect CsI scintillator detector	100 μm	56 μm	NU	1.8	Mo/Mo	25 mAs 50 mAs 70 mAs 100 mAs 140 mAs	27 kVp	CDMAM [46]/ gold disks, diameters 0.1–0.5 mm	NS	0.10–0.50 mm (diameter)	CDD and COR

*NA* not applicable, *NU* not used, *NS* not specified, *Mo* molybdenum, *Rh* rhodium, *W* wolfram, *Al* aluminium, *Ag* silver, *PMMA* polymethyl methacrylate, *CsI* caesium iodide, *AEC* automatic exposure control, *ACR* American College of Radiology, *AGD* average glandular dose, *CO* calcium oxalate, *HA* hydroxyapatite, *PI* performance index, *IQF* image quality figure, *CCD* contrast detail detection, *CNR* contrast-to-noise ratio, *COR* correct observation ratio *Objective measurements

Detection was studied under varying current—time products (mAs), tube potentials (kVp), anode/filter combinations, detector technologies and magnification/zoom factors, as indicated in Table 1. One study [36] used the ACR phantom [45], two studies [38, 40] the CDMAM phantom [46], one study [37] an aluminium square of 0.2-mm thickness embedded in polymethyl methacrylate (PMMA) and one study [39] a simulated phantom utilising the MASTOS model [47]. In the latter, the simulation was performed for a range of glandularities. The sizes of microcalcifications varied according to what was available in the phantoms. Outcome measures for detection also varied: RANK sum score for visibility [36], normalised performance index (PI) [37], image quality figure (IQF) [38], contrast-detail detection (CDD) [38, 40], contrast-to-noise ratio (CNR) [39] and correct observation ratio (COR) [40]. Comments on the main findings relating to these quantities are listed in Table 2.

**Table 2:** Results from the experimental phantom studies

Author	Results
Alkhalifah et al [36]	The choice of tube potential (kVp) did not have a statistically significant effect on scores for either magnification or zoom. Magnification: Rank sum scores were 32.58, 22.38 and 36.55 for target/filter Mo/Mo, Mo/Rh and Rh/Rh respectively. Zoom: Rank sum scores were 29.10, 39.15 and 23.25 for target/filter Mo/Mo, Mo/Rh and Rh/Rh respectively. Target/filter Mo/Mo and Rh/Rh: Rank sum scores and mean scores are significantly better (*p* < 0.05) for magnification than for zoom. Target/filter Mo/Rh: Rank sum scores and mean scores are significantly better (*p* < 0.05) for zoom than for magnification.
Egan et al [37]	PI was higher for mass detection than detection of microcalcifications when standardised AEC was utilised. Adjusting the exposure factors accordingly increased the normalised PI and the detection of microcalcifications for all three imaging systems. The scanning photon counting system had normalised PI comparable to the conventional magnification imaging systems for detection of microcalcifications when optimised exposure factors were used. These results apply to three breast thicknesses and all three detector technologies in the experiment.
Vahey et al [38]	Magnification: IQF = 1.28 ± 0.33 Zoom: IQF = 1.91 ± 0.47, *p* = 0.055 The difference between IQF for magnification and zoom was not statistically significant. CDD: Contrast-detail detection for most disk diameters under or equal to 0.63 mm and all disk diameters under 0.20 mm are statistically significantly better for magnification than for zoom. *p* values are 0.044, 0.026, 0.035, 0.217, 0.101, 0.018, 0.005 and 0.005 for diameters 0.63 mm, 0.50 mm, 0.40 mm, 0.31 mm, 0.25 mm, 0.20 mm, 0.16 mm and 0.13 mm, respectively. For disk diameters 0.80–2.00 mm the differences are not statistical significant.
Koutalonis et al [39]	A microcalcification is visible if CNR > 1 CNR increases when magnification/zoom factor increases. CNR increases when the size of the microcalcification increases. CNR decreases when glandularity increases. CNR for magnification > CNR for zoom for all magnification zoom factors, all glandularities and for all sizes of microcalcifications. The relative difference is largest for the smallest microcalcifications and for the highest glandularity. Microcalcifications of radii 0.05 mm or smaller are only visible with magnifications and magnification factors > 1.7 Microcalcifications of radii 0.1 mm are only visible with magnifications and magnification factors > 1.4 or zoom factors > 1.7 Microcalcifications of radii 0.25 mm and larger are visible in both magnification and zoom
Hermann et al [40]	Contrast-detail detection improves when current-time product (mAs) increases. Correct observation ratio (COR) of simulated microcalcifications of diameters 0.10–0.50 mm improves with increased mAs; COR = 0.55, 0.81, 0.83, 0.90 and 0.95 for 25 mAs, 50 mAs, 70 mAs, 100 mAs and 140 mAs, respectively.

Diagnostic test studies [41–44] reported clinical characteristics and diagnostic accuracy data for magnification and zoom at a threshold equal to or equivalent to BIRADS ≥ 4a [48], see Table 3. The ranges of sensitivity and specificity were 85–100% and 50–57%, respectively, for magnification. For zoom, the ranges of sensitivity and specificity were 59–98% and 43–62%, respectively. The total number of true positives, false positives, false negatives and true negatives from each diagnostic test study is listed in Table 4.

**Table 3:** Retrospective diagnostic test studies: Study characteristics, technical characteristics and clinical characteristics

Author	Year	Location	Study period	No. of readers	Reader experience, years/mean/SD/range	Apparatus/detector technology	Pixel size/pixel depth	Effective pixel size magnification	Magnification factor	Zoom factor	No. of patients/lesions/images	Patient age, years/mean/SD/range	Case characteristics and reference standard	Pre-test probability (%)
Fallenberg et al [41]	2014	Germany	Jan. 2000–Dec. 2007	6	8.6/6.2/[1.5–19]	GE Senographe 2000D/indirect CsI scintillator detector	100 μm/NS	57 μm	1.75	2.0	100/100/NS	57.3/8.9/[37–76]	*Positive cases*: 35 malign cases with biopsy as reference standard: 12 invasive carcinomas, 23 DCIS. *Negative cases*: 65 cases: 11 nodular fibrosis, 16 fibrocystic mastopathy, 1 radial scar, 1 dysplastic hyaline scar, 3 papilloma, 1 fatty tissue, all biopsy proven, and 32 benign with 2-year follow-up as reference standard.	35.0
Moraux-Wallyn et al [42]	2010	France	Feb. 2005–Mar. 2007	2	NS/NS/NS	Siemens Mammomat Novation DR/direct amorphous Selenium detector	70 μm/NS	39 μm	1.8	1.8	82/88/328	NS/NS/NS	*Positive cases*: 19 malign cases, including ductal carcinoma, invasive lobular carcinoma and DCIS .10 atypical cases, including atypical lobular or atypical hyperplasia, lobular carcinoma in situ, all with biopsy as reference standard. *Negative cases*: 59 benign cases, including benign with biopsy as reference standard and benign with two-year follow-up as reference standard.	33.1
Kim et al [43]	2010	South Korea	Oct. 2006–Feb. 2008	3	7.0/4.4/[4–12]	Lorad-Hololic Selenia/direct amorphous Selenium detector	70 μm/14 bits	39 μm	1.8	2.0	185/185/740	49.9/NS/[27–69]	*Positive cases*: 43 malign cases with biopsy as reference standard. *Negative cases*: 142 benign cases with biopsy as reference standard.	23.2
Kim et al [44]	2009	South Korea	May 2005–Oct. 2006	3	6.3/4.9/[1–10]	Lorad- Hololic Selenia/direct amorphous Selenium detector	70 μm/ 12 bits	39 μm	1.8	1.3	111/ 120/ 480	NS/ NS/ NS	*Positive cases:* 28 malign cases with biopsy as reference standard. *Negative cases*: 51 benign cases with biopsy as reference standard, 41 benign cases with at least two-year follow-up as reference standard.	23.3

*NS* not specified, *DCIS* ductal carcinoma in situ

**Table 4:** Numbers extracted from the retrospective diagnostic test studies: number of true positives (TP), number of false positives (FP), number of false negatives (FN) and number of true negatives (TN) from zoom and magnification

	Zoom				Magnification			
Author	TP	FP	FN	TN	TP	FP	FN	TN
Fallenberg et al [41]	124	164	86	226	178	167	32	223
Moraux-Wallyn et al [42]	53	61	5	46	58	50	0	67
Kim et al [43]	119	184	10	242	119	211	10	215
Kim et al [44]	73	104	11	172	77	119	7	157

**Fig. 2:**
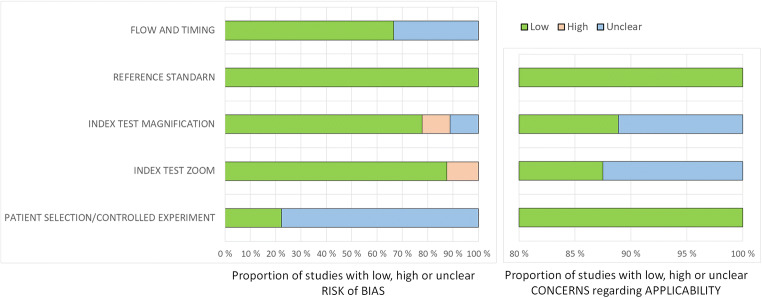
Risk of bias and applicability. Grouped bar charts showing risk of bias (left) and concerns regarding applicability (right) for the included studies, using the QUADAS2 domain for the diagnostic test studies, and the modified version of QUADAS2 for the phantom studies

Figure 2 shows the summarised result of the quality assessment of the included studies. One of the studies came out as at ‘high risk of bias’ because the readers were not blinded with regard to the use of magnification and zoom. We also determined that it was ‘unclear’ whether some of the studies met certain quality criteria. The specific reasons were as follows: two gold standards were used in three out of four diagnostic test studies, the retrospective design of certain diagnostic test studies, and that three of the phantom studies [37, 39, 40] did not state standard deviations, 95% confidence intervals or *p* values.

### Detection of microcalcifications

Findings concerning the detection of microcalcifications from the phantom studies were summarised in Table 2.

Detection of the smallest microcalcifications (diameters < 200 μm) was higher when using magnification than zoom, whereas detection was more comparable for larger microcalcifications [38, 39]. According to Vahey et al [38], there are also statistically significant differences in detection in the range of 200–630 μm. Koutalonis et al [39] found that microcalcifications of radii 50–100 μm are only visible when utilising magnification.

The detection of microcalcifications rose with increased current-time product (mAs) [40], decreased glandularity and increased magnification or zoom factor [39]. Changing the tube voltage (kVp) while current-time product is controlled by automatic exposure control (AEC) did not have a statistically significant effect on the detection of microcalcifications regardless of whether the magnification or zoom was used [36]. Magnification yields higher detection of microcalcifications for anode/filter combinations Mo/Mo and Rh/Rh, while zoom yields higher detection for the anode/filter combination Mo/Rh [36].

According to Egan et al [37], normalised PI was higher for mass detection than microcalcification detection when using standard AEC. Optimising the exposure factors improved detection both for conventional FFDM magnification mammography and photon counting FFDM without magnification, and the value of normalised PI for photon counting FFDM was comparable to the conventional FFDM with magnification [37].

### Diagnosing microcalcifications

**Fig. 3:**
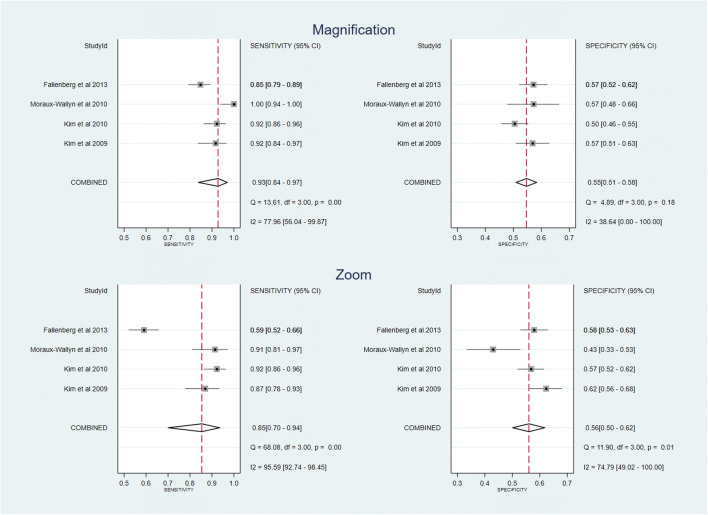
Coupled forest plots of pooled sensitivity and specificity for diagnosing microcalcifications using magnification images (above) and zoom (below). The squares represent the sensitivities and specificities for individual studies, while the horizontal lines plot their 95% confidence interval. The pooled sensitivities and specificities are indicated with a red dotted vertical line and a diamond, while the horizontal size of the diamonds indicates their 95% confidence interval. Results from the heterogeneity tests are also listed in the lower right corner of the plots

Coupled forest plots of sensitivity and specificity for magnification and zoom are shown in Fig. 3. Pooled sensitivity was 0.93 (95% CI 0.84–0.97) and 0.85 (95% CI 0.70–0.94) for magnification and zoom respectively. The pooled specificity was similar for both 0.55 (95% CI 0.51–0.58) and 0.56 (95% CI 0.50–0.62) for magnification and zoom, respectively.

A likelihood ratio test was performed, comparing a bivariate model without a covariate for diagnostic test type (magnification or zoom) with a bivariate model that included a covariate for diagnostic test type and assumed equal variance for each diagnostic test. There was no statistical evidence that sensitivity and/or specificity differed between magnification and zoom (*p* = 0.42). Likelihood ratio tests for sensitivity and specificity alone gave non-significant differences for both sensitivity (*p* = 0.20) and specificity (*p* = 0.57) between magnification and zoom.

The *Q* test did not indicate any heterogeneity among the observations of specificities from individual studies of magnification (*Q* = 4.89, *p* = 0.18), and the *I*^2^ test confirmed that heterogeneity was not significant (*I*^2^ = 38.64%). However, we did detect heterogeneity in the cases of sensitivity of magnification and both sensitivity and specificity of zoom (*Q* tests yielded *p* < 0.05) and could be considerable (*I*^2^ values = 77.96%, 95.59% and 74.79%, respectively).

## Discussion

In this systematic review, we have compared the use of magnification and zoom for detecting and diagnosing microcalcifications. Our review of phantom studies found that the size of microcalcifications, exposure factors and detector technology determine whether or not digital zoom is equivalent to magnification in the detection of microcalcifications. Our meta-analysis of sensitivity and specificity from the diagnostic test studies found high sensitivities for both magnification and zoom 0.93 (95% CI 0.84–0.97) and 0.85 (95% CI 0.70–0.94), respectively, but low specificities 0.55 (95% CI 0.51–0.58) and 0.56 (95% CI 0.50–0.62), respectively. No statistically significant differences were found between the sensitivities or the specificities.

Malign microcalcifications are likely to occur in the range 50–500 μm, while benign calcifications are often larger than 1 mm [1]. The observed differences in detection between magnification and zoom apply to the size corresponding to the smallest malignant microcalcifications. Other phantom studies [8, 9] revealed that pixel sizes below 100 μm enhance the visual perception of small objects corresponding to typical microcalcifications, and detection of microcalcifications increases as pixel size decreases. A detector pixel size of 100 μm, as used in three out of five phantom studies in our review, would then require magnification for a more optimal effective pixel size. A detector pixel size of 100 μm was also used in one of the diagnostic test studies [41]. This study showed that microcalcifications were more visible and more microcalcifications were detected when using geometric magnification than digital zoom.

Optimising the exposure factor also improves detection of microcalcifications: An increase in mAs/ESAK improved detection [40], in line with another study, which demonstrated that reduced noise improved reader performance when detecting microcalcifications [10]. Increasing tube potential (kVp) decreases contrast, but a phantom study [36] could not demonstrate a statistically significant decrease in detection. However, zoom yielded better detection using anode/filter combination Mo/Rh, while magnification yielded better detection for Mo/Mo and Rh/Rh [36]. The author of this study proposed further investigations on how spectra of x-rays influence the visibility of structures [36].

Newer photon-counting detector technology in combination with optimised exposure factors may generate non-magnified images with microcalcification detection equivalent to magnified images from FFDM with flat panel detectors [37]. Standard AEC seems to be best suited to imaging masses, and exposure factors should always be optimised for the purpose of detecting microcalcifications, whether you use magnification or photon counting technology. However, this was not tested on different sizes of microcalcifications.

Our meta-analysis of sensitivity and specificity for magnification and zoom based on diagnostic test studies did not find any statistically significant differences. A partial explanation may be that most of the diagnostic studies included were performed using detectors of smaller pixel sizes (70 μm) than most of the phantom studies, which offer a better visual perception of microcalcifications also for zoomed images. The absence of any significant difference is in agreement with earlier studies based on digitised analogue images [19] or hard-copy prints of digital images [20], which suggests that zooming provides valuable information about microcalcifications [19]. If zoom could replace magnification in recalls due to suspicion of microcalcifications, it would reduce AGD, number of potentially painful compressions and examination time, thereby improving workflow [20].

The meta-analysis revealed heterogeneity between the diagnostic test studies. The fact that no statistically significant differences were found between the sensitivity for magnification and zoom could also be due to heterogeneity implying broader 95% confidence intervals for the pooled value and a non-significant *p* value and obscuring real differences. Some conditions cause more subtle microcalcifications than others [1, 2], and differences in patient age, case characteristics and pre-test probabilities, as listed in Table 3, may contribute to heterogeneity. Differences in imaging detectors introduce differences in image noise and resolution and could also contribute to heterogeneity. According to the Nyquist sampling theorem, objects smaller than twice the detector pixel size will either not be visualised or will be incorrectly visualised due to aliasing [10]. Varying reader experience is also a potential factor; a study has shown that experienced readers perform better in the detection of microcalcifications than inexperienced readers [14].

The retrospective design of the diagnostic test studies and different reference standards for benign lesions might be a limitation in this review. The studies used images available from clinical practice where both magnification and conventional FFDM were available; this may have led to a selection bias in the sensitivity and specificity of the individual studies. It should be mentioned that some of the experimental phantom studies did not provide standard deviations and/or confidence intervals. More diagnostic studies, studies using newer detector technology and studies considering post-processing of magnified and zoomed images would have strengthened this review. The phantom images in the included experimental studies have a uniform background in contrast to the background of real mammograms where the anatomic noise could be a limiting factor [49]. Experimental studies comparing magnification and zoom with anthropomorphic breast phantoms could be an option for further investigations. Nevertheless, this systematic review provides an overview of studies using FFDM magnification and zoom to compare detection and diagnosing of microcalcifications in diagnostic mammography.

In conclusion, zoom may be equivalent to magnification in many cases given that optimised procedures and newer detector technologies are now available. This finding has the potential to reduce AGD and improve examination workflow. Both diagnostic test studies and phantom studies using newer detectors would contribute additional knowledge on this topic.

## Electronic supplementary material

ESM 1 (DOCX 269 kb)

## Abbreviations

AGD: Average glandular dose
CI: Confidence interval
CNR: Contrast-to-noise ratio
FFDM: Full-field digital mammography
PRISMA: Preferred Reporting Items for Systematic Reviews and Meta-Analyses
QUADAS-2: Quality Assessment of Diagnostic Accuracy Studies-2
SNR: Signal-to-noise ratio
