# Supplementary material for: Digital zoom of the full-field digital mammogram versus magnification mammography: a systematic review
Source: Eur Radiol. 2020 Mar 28;30(8):4223–33. doi: 10.1007/s00330-020-06798-6 (PMC7338280; doi:10.1007/s00330-020-06798-6)
Supplement: Supplementary file 1 — (DOCX 269 kb) [file 330_2020_6798_MOESM1_ESM.docx]

# Search strategy

A systematic literature search was conducted in Embase (Ovid), MEDLINE (Ovid), CINAHL (EBSCO), Engineering Village: Compedex and Web of Science.

The search included controlled vocabulary terms and free-text terms in the following combination: (mammography OR microcalcification) AND (digital magnification OR geometric magnification), as indicated in table 5. Each search was customised for the particular database by using unique terms from the index, in addition to identified synonymous free-text terms including proximity operators, when appropriate. We searched Web of Science by topic. The search strategies for the databases are shown in tables 6-10.

**Table 5. Search strategy.**

| **Patient/problem:**  **Mammography or microcalcification** | **Intervention:**  **Digital magnification** | **Comparison:**  **Geometric magnification** |
| --- | --- | --- |
| mammography  OR  breast phantom  OR  breast density  OR  microcalcifications | digital zoom  OR  digital magnification  OR  electronic zoom | radiographic magnification  OR  geometric magnification  OR  air gap |

**Table 6. Search strategy in Embase.**

Database: **Embase** <1980 to 2019 Week 36>

Search Strategy:

--------------------------------------------------------------------------------

1 exp mammography/ (52721)

2 mammogra*.ti,ab,kw. (40152)

3 (breast adj3 phantom*).ti,ab,kw. (1155)

4 (breast adj3 density).ti,ab,kw. (3457)

5 (mammogra* adj3 density).ti,ab,kw. (2409)

6 CDMAM.ti,ab,kw. (85)

7 calcification*.ti,ab,kw. (69041)

8 exp breast calcification/ (3047)

9 microcalcification*.ti,ab,kw. (4899)

10 1 or 2 or 3 or 4 or 5 or 6 or 7 or 8 or 9 (128863)

11 (digital adj3 zoom*).ti,ab,kw. (57)

12 (digital adj3 magnification*).ti,ab,kw. (195)

13 (electronic adj5 zoom*).ti,ab,kw. (25)

14 (electronic adj5 magnification*).ti,ab,kw. (82)

15 exp digital mammography/ (2389)

16 Full-field digital mammogra*.ti,ab,kw. (825)

17 FFDM.ti,ab,kw. (615)

18 11 or 12 or 13 or 14 or 15 or 16 or 17 (3149)

19 (radiograph* adj5 magnification*).ti,ab,kw. (454)

20 exp magnification radiography/ (476)

21 air gap.ti,ab,kw. (877)

22 exp air/ (44038)

23 (geometr* adj5 magnification*).ti,ab,kw.(103)

24 19 or 20 or 21 or 22 or 23 (45653)

25 18 or 24 (48727)

26 10 and 25 (2970)

**Table 7. Search strategy in Medline.**

Database: **Ovid MEDLINE**(R) and Epub Ahead of Print, In-Process & Other Non-Indexed Citations and Daily <1946 to September 10, 2019>

Search Strategy:

--------------------------------------------------------------------------------

1 exp Mammography/ (29034)

2 mammogra*.ti,ab,kw. (31321)

3 (breast adj3 phantom*).ti,ab,kw. (937)

4 (breast adj3 density).ti,ab,kw. (2333)

5 (mammogra* adj3 density).ti,ab,kw. (1713)

6 CDMAM.ti,ab,kw. (63)

7 Calcification*.ti,ab,kw. (52902)

8 exp Calcinosis/ (43692)

9 microcalcification*.ti,ab,kw. (3553)

10 1 or 2 or 3 or 4 or 5 or 6 or 7 or 8 or 9 (112982)

11 (digital adj3 zoom*).ti,ab,kw. (40)

12 (digital adj3 magnification*).ti,ab,kw. (160)

13 (electronic adj5 zoom*).ti,ab,kw. (19)

14 (electronic adj5 magnification*).ti,ab,kw. (61)

15 FFDM.ti,ab,kw. (417)

16 Full-field digital mammogra*.ti,ab,kw. (631)

17 11 or 12 or 13 or 14 or 15 or 16 (955)

18 exp Radiographic Magnification/ (965)

19 (radiograph* adj5 magnification*).ti,ab,kw. (488)

20 (geometr* adj5 magnification*).ti,ab,kw. (123)

21 air gap.ti,ab,kw. (815)

22 exp Air/ (25502)

23 18 or 19 or 20 or 21 or 22 (27559)

24 17 or 23 (28441)

25 10 and 24 (883)

**Table 8. Search strategy in Cinahl.**

September 10, 2019

| **Search ID#** | **Search Terms** | **Results** |
| --- | --- | --- |
| S25 | \| S10 AND S24 \|  \| \| --- \| --- \| | (215) |
| S24 | \| S17 OR S23 \|  \| \| --- \| --- \| | (2,380) |
| S23 | \| S18 OR S19 OR S20 OR S21 OR S22 \|  \| \| --- \| --- \| | (2,138) |
| S22 | \| geometr* N5 magnification* \|  \| \| --- \| --- \| | (4) |
| S21 | \| (MH "Air+") \|  \| \| --- \| --- \| | (1,489) |
| S20 | \| air gap* \|  \| \| --- \| --- \| | (480) |
| S19 | \| Radiograph* N5 Magnification* \|  \| \| --- \| --- \| | (177) |
| S18 | \| (MH "Radiographic Magnification") \|  \| \| --- \| --- \| | (125) |
| S17 | \| S11 OR S12 OR S13 OR S14 OR S15 OR S16 \|  \| \| --- \| --- \| | (253) |
| S16 | \| Full-field digital mammogra* \|  \| \| --- \| --- \| | (190) |
| S15 | \| "FFDM" \|  \| \| --- \| --- \| | (125) |
| S14 | \| electronic N5 magnification* \|  \| \| --- \| --- \| | (8) |
| S13 | \| electronic N5 zoom* \|  \| \| --- \| --- \| | (2) |
| S12 | \| digital N3 magnification* \|  \| \| --- \| --- \| | (36) |
| S11 | \| digital N3 zoom \|  \| \| --- \| --- \| | (1) |
| S10 | \| S1 OR S2 OR S3 OR S4 OR S5 OR S6 OR S7 OR S8 OR S9 \|  \| \| --- \| --- \| | (19,693) |
| S9 | \| (MH "Calcinosis") \|  \| \| --- \| --- \| | (5,765) |
| S8 | \| calcinosis \|  \| \| --- \| --- \| | (5,944) |
| S7 | \| "microcalcification*" \|  \| \| --- \| --- \| | (4,549) |
| S6 | \| CDMAM \|  \| \| --- \| --- \| | (7) |
| S5 | \| mammogra* N3 density \|  \| \| --- \| --- \| | (688) |
| S4 | \| breast N3 density \|  \| \| --- \| --- \| | (1,061) |
| S3 | \| breast N3 phantom* \|  \| \| --- \| --- \| | (98) |
| S2 | "mammogra*" | (13,624) |
| S1 | (MH "Mammography") | (10,838) |

**Table 9. Search strategy in Web of Science.**


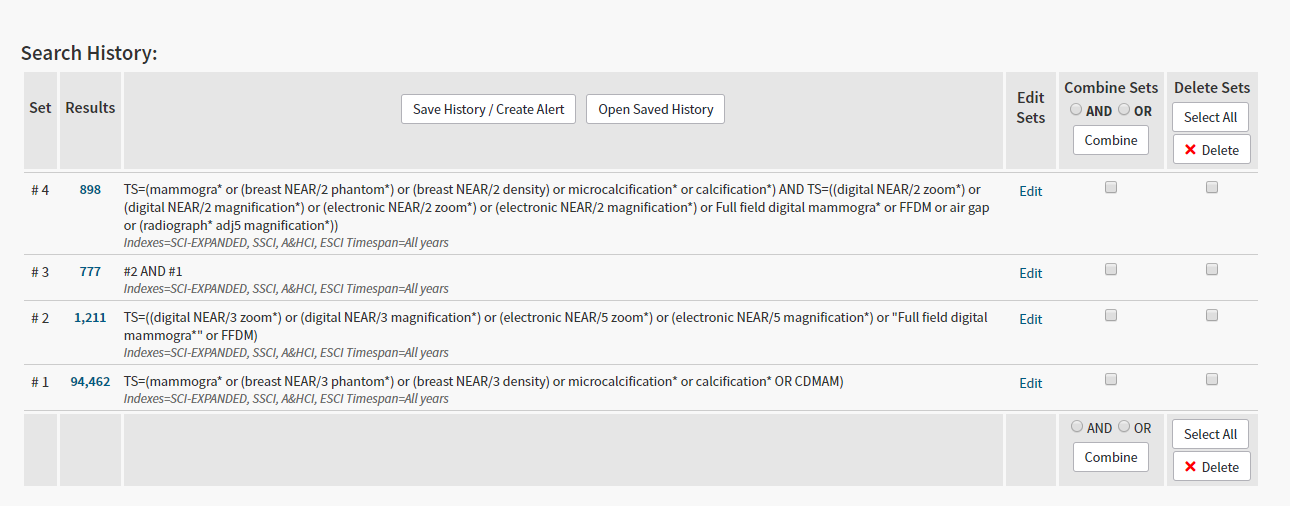


**Table 10. Search strategy in Engineering Village.**


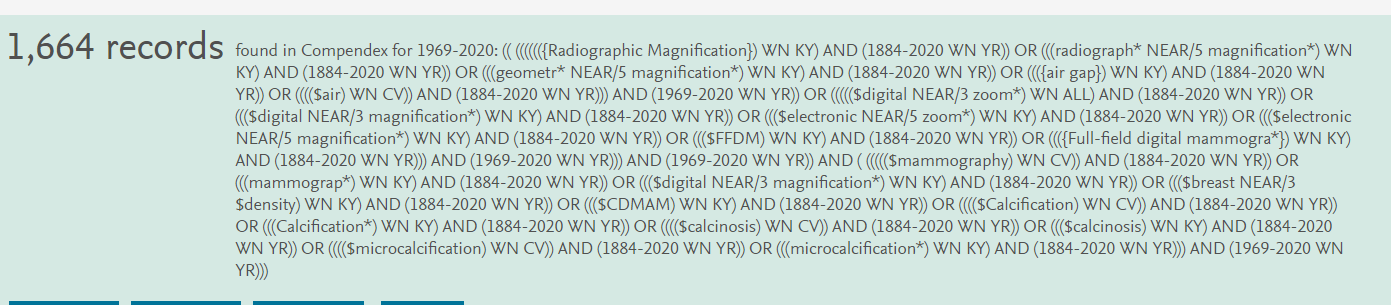


# Grey literature search

The following sources of grey literature were searched:

- international/national guidelines from ICRP, IAEA, ISRRT, NCRP, NHSBSP, EU, the Nordic countries and the guidelines listed in Box 7.2.d in Cochrane Handbook for Systematic reviews of Diagnostic Test Accuracy
- conference presentations, abstracts and posters from RSNA, ECR, Nordic Congress of Radiology and International workshop on Digital mammography and the conference abstract sources listed in Box 7.2.g in Cochrane Handbook for Systematic reviews of Diagnostic Test Accuracy
- reference lists in included studies and citations
- grey literature databases: http://www.greylit.org/ http://www.greynet.org/opensiglerepository.html, www.ntis.gov/
- and other free databases: www.who.int/hinari/en/ , www.inasp.info/file/68/about-inasp.html, www.eifl.net/cps/sections/about
